# A Reduced Astrocyte Response to β-Amyloid Plaques in the Ageing Brain Associates with Cognitive Impairment

**DOI:** 10.1371/journal.pone.0118463

**Published:** 2015-02-23

**Authors:** Ryan Mathur, Paul G. Ince, Thais Minett, Claire J. Garwood, Pamela J. Shaw, Fiona E. Matthews, Carol Brayne, Julie E. Simpson, Stephen B. Wharton, on behalf of the MRC Cognitive Function and Ageing Neuropathology Study Group

**Affiliations:** 1 Sheffield Institute for Translational Neuroscience, University of Sheffield, Sheffield, England, United Kingdom; 2 Institute of Public Health, University of Cambridge, Cambridge, England, United Kingdom; 3 MRC Biostatistics Unit, Cambridge, England, United Kingdom; Torrey Pines Institute for Molecular Studies, UNITED STATES

## Abstract

**Aims:**

β-amyloid (Aβ) plaques are a key feature of Alzheimer’s disease pathology but correlate poorly with dementia. They are associated with astrocytes which may modulate the effect of Aβ-deposition on the neuropil. This study characterised the astrocyte response to Aβ plaque subtypes, and investigated their association with cognitive impairment.

**Methods:**

Aβ plaque subtypes were identified in the cingulate gyrus using dual labelling immunohistochemistry to Aβ and GFAP^+^ astrocytes, and quantitated in two cortical areas: the area of densest plaque burden and the deep cortex near the white matter border (layer VI). Three subtypes were defined for both diffuse and compact plaques (also known as classical or core-plaques): Aβ plaque with (1) no associated astrocytes, (2) focal astrogliosis or (3) circumferential astrogliosis.

**Results:**

In the area of densest burden, diffuse plaques with no astrogliosis (β = -0.05, p = 0.001) and with focal astrogliosis (β = -0.27, p = 0.009) significantly associated with lower MMSE scores when controlling for sex and age at death. In the deep cortex (layer VI), both diffuse and compact plaques without astrogliosis associated with lower MMSE scores (β = -0.15, p = 0.017 and β = -0.81, p = 0.03, respectively). Diffuse plaques with no astrogliosis in layer VI related to dementia status (OR = 1.05, p = 0.025). In the area of densest burden, diffuse plaques with no astrogliosis or with focal astrogliosis associated with increasing Braak stage (β = 0.01, p<0.001 and β = 0.07, p<0.001, respectively), and ApoEε4 genotype (OR = 1.02, p = 0.001 and OR = 1.10, p = 0.016, respectively). In layer VI all plaque subtypes associated with Braak stage, and compact amyloid plaques with little and no associated astrogliosis associated with ApoEε4 genotype (OR = 1.50, p = 0.014 and OR = 0.10, p = 0.003, respectively).

**Conclusions:**

Reactive astrocytes in close proximity to either diffuse or compact plaques may have a neuroprotective role in the ageing brain, and possession of at least one copy of the *ApoEε4* allele impacts the astroglial response to Aβ plaques.

## Introduction

Neuropathologically Alzheimer’s disease (AD) is a progressive neurodegenerative disorder characterised by β-amyloid (Aβ) plaques (diffuse and compact dense-core) and intracellular tangles of hyperphosphorylated tau [1]. Aβ plaque formation is thought to progress from diffuse through to compact [2,3]. The relative frequency of the plaque subtypes changes during the progression of AD, with diffuse Aβ plaques being prevalent in the preclinical stages and compact plaques increasing in frequency as the disease progresses [4,5]. The Medical Research Council’s population-based Cognitive Function and Ageing Study (CFAS) has shown that Alzheimer-type pathology is the most common pathology associated with dementia in the ageing population [6], but that there is considerable overlap in the burden of plaques and tangles between individuals with or without dementia, especially in the oldest old [7,8], suggesting other factors contribute to the progression of cognitive decline.

Astrocytes, the most abundant glial cell, play a critical role in neuronal support and in maintaining homeostasis within the central nervous system (CNS) [9]. Activated glia surround and infiltrate Aβ plaques in AD [10–12], however their exact role in the pathogenesis of age-related neuropathology remains unknown. While astrocytes have been shown to play a significant role in the degradation and clearance of Aβ suggesting a neuroprotective role [13], other studies have shown astrocyte activation results in the production of critical inflammatory mediators, suggesting they play a detrimental role in the progression of age-related neurodegenerative pathology [14]. Reactive astrocytes up-regulate glial fibrillary acidic protein (GFAP) expression in response to CNS insults [15,16]. Astrogliosis and astrocyte dystrophy are prominent features of several dementia pathologies, including AD and frontotemporal dementia, where the degree of astrocyte degeneration correlates with the severity of dementia [17]. Astrogliosis occurs at early stages of AD pathogenesis and treatment of cultured astrocytes with aggregated Aβ or with amyloid isolated from human AD brains has been shown to trigger astrogliosis [18–20]. Recent studies have further characterised the astroglial response in AD, demonstrating an increase in plaque-associated GFAPα and GFAPδ isoforms, and although the number of astrocytes expressing the GFAP(+1) isoform correlates with AD progression, they are not associated with plaques [12]. Possession of the *ApoEε4* allele, a major genetic risk factor for AD [21], is associated with an increased cortical Aβ plaque burden [22–24] and astrocyte dysfunction [25]. ApoE4, primarily expressed by astrocytes in the brain, plays a role in the metabolism of amyloid [26,27], and has been shown to promote Aβ deposition [28].

The CFAS neuropathology cohort is population-based thus allowing unbiased assessment of pathologies in brain ageing and their relationships to cognitive impairment [6–8]. We have previously characterised the astrocyte phenotype in the CFAS cohort and demonstrated increased GFAP immunoreactivity associated with increasing Braak and Braak neurofibrillary tangle stage with some, but not all, Aβ plaques associated with GFAP^+^ astrocytes [29]. We hypothesised that the astrocyte response to Aβ deposits in the cingulate gyrus may modulate the effect of the amyloid plaque on surrounding brain tissue, and therefore on cognition. This region was selected as it contributes to spatial learning and memory [30], is associated with a high prevalence of Aβ pathology. It is involved in the intermediate stages of Aβ progression (Aβ phase 3/5), [31], is involved in the limbic stage of neurofibrillary tangle progression (Braak and Braak stage III-IV) and has projections to the entorhinal cortex, the area with the earliest NFT formation [32], and presents with metabolic and vascular changes before the development of AD [33,34]. Therefore the aim of this study was to examine the variation in the astrocyte response associated with both diffuse and compact Aβ plaques in the cingulate gyrus, and investigate their association with Braak and Braak stage, cognitive impairment, dementia status and ApoE genotype.

## Materials and Methods

### Human CNS cases

Human CNS material was obtained from the Medical Research Council Cognitive Function and Ageing Study (MRC CFAS) autopsy cohort, which has been described in detail previously [35,36]. Individuals selected for the assessment interviews were approached by a trained liaison officer for brain donation in each centre, who discussed the donation programme with the respondent and his or her family or carers, as appropriate. When an individual died and the research team was notified, the next of kin was approached to give consent for brain donation and retention. Limited or full necroscopy then proceeded if all permissions were obtained. Multi-centre research ethics committee (REC) approval for the current study was obtained from Cambridgeshire 1 Research Ethics Committee (REC reference number 10/H0304/61).

The study used all of the cases derived from one of the CFAS centres (Cambridge), thereby maintaining the unbiased, population-based nature of the study. Cortical blocks were sampled within 4–6 weeks following a standard protocol [37], from 109 formalin-fixed cases. Neuropathological lesions were assessed as part of the core CFAS neuropathology study using a modified protocol from the Consortium to Establish a Registry of Alzheimer’s Disease (CERAD) [38] (wwws.cfas.ac.uk). Braak and Braak staging was assessed by analysis of AT8 immunostaining of neurofibrillary tangles in the hippocampus and isocortical regions [32,39]. The cases were categorised into groups representing entorhinal stages (Braak stages 0–2; 27 cases), limbic stages (Braak stages 3–4; 50 cases) and isocortical stages (Braak stages 5–6; 22 cases). ApoE genotype was previously determined in the cohort [40,41].

Individuals in the study were regularly interviewed and underwent Geriatric Mental State-Automated Geriatric Examination for Computer-Assisted Taxonomy (GMS-AGECAT), Cambridge Mental Disorders of the Elderly Examination (CAMDEX), and mini mental state examination (MMSE) [7,8]. Dementia status at death was determined on the basis of all information available for each participant, as previously described [8,35]. Within this cohort, 68 participants had dementia, 39 had no dementia and 2 participants had an unknown dementia status at death due to the lack of information in the years preceding death (Table 1). Twenty-one participants with dementia and 9 participants with no dementia possessed at least one *ApoEε4* allele.

**Table 1:** Demographic and cognitive profile of cases, according to dementia status.

	No dementia (n = 39)	Dementia (n = 68)
Sex (male:female)	16:23	22:46
Age at death (y)*	84 (76–88)	89 (84–93)
Years since last cognitive assessment^a^	1.5 (1.0–1.9)	1.7 (0.8–3.0)
MMSE at last assessment*	24 (20–27)	14 (6–20)

^a^median (interquartile range)

### Immunohistochemistry

Immunohistochemistry of formalin-fixed, paraffin-embedded sections (5μm) from the cingulate cortex was performed using a standard avidin-biotin complex (ABC) method. Sections were deparaffinised, rehydrated to water and endogenous peroxidase activity quenched by placing the sections in 0.3% H_2_O_2_/methanol for 20min at room temperature (RT). Sections were subjected to antigen retrieval (0.01M tri-sodium citrate pH6.5, microwave 10min) followed by formic acid pre-treatment for 60min at RT. Following incubation with 1.5% normal serum for 30min at RT, the sections were incubated with anti-Aβ (Clone 6F/3D; DakoCytomation, UK) [42] at the optimal antibody dilution of 1:200 for 60min at RT. To visualise antibody binding, the horse-radish peroxidase avidin biotin complex was used (Vectastain Elite kit, Vector Laboratories, UK) with 3,3’-diaminodenzidine (DAB) as the chromagen (Vector Laboratories, UK; brown). Following incubation with the avidin-biotin blocking kit (Vector Laboratories, UK), sections were incubated overnight at 4°C with anti-GFAP (1:500; DakoCytomation, UK) [43], followed by the alkaline-phosphatase-conjugated avidin-biotin complex (Vectastain Elite kit, Vector Laboratories, UK), developed with alkaline phosphatase substrate 1 (Vector Laboratories, UK; red) and lightly counterstained with Mayer’s haematoxylin. Negative controls, either omission of the primary antibody or isotype controls, were included in every run.

### Quantitative neuropathological analysis

Assessment of Aβ and GFAP immunoreactivity was conducted using a Nikon Eclipse Ni-U microscope and Nikon DS-Ri1 camera with NIS-Elements BR 4.20.01 64-bit microscope imaging software (Nikon, UK). Aβ plaques were subtyped based on the type of plaque (diffuse or compact) and the surrounding astrocyte reaction. Compact plaques are spherical in shape and are characterised by a dense central core of Aβ surrounded by a less compact peripheral halo, in contrast to the diffuse plaques which are usually not spherical and stain weakly for Aβ [44]. Compact plaques may be associated with tau-positive dystrophic neurites, which are then referred to as neuritic plaques. However, triple labelling with Aβ, GFAP and tau was not performed, therefore neuritic plaques containing dystrophic neurites were not assessed as part of this study. The frequency and grade (none, mild, moderate or severe) of both diffuse and compact Aβ plaques were assessed in single Aβ immunostained cingulate gyrus sections, based on the CERAD protocol [38].

Three subtypes were defined for both diffuse and compact plaques: (1) Aβ plaque with no associated astrocytes, (2) focal astrogliosis and (3) circumferential astrogliosis. Focal astrogliosis was defined as reactive astrocytes directly in contact with a plaque solely on one of its borders. Circumferential astrogliosis was defined as reactive astrocytes directly in contact with and completely surrounding a plaque. Areas of astrogliosis remote from plaques were also identified and defined as small (less than two distinct GFAP^+^ astrocytes) and large (greater than or equal to three distinct GFAP^+^ astrocytes). The number of each plaque subtype was quantitated in the area with the densest of amyloid burden under the 10x objective (field area 1275 x 925 μm^2^). Micro-plaques (<10μm in diameter) and plaques in layer I of the cortex were ignored and did not contribute to the count. Using an identical method, the number of each plaque subtype was also assessed in four fields of the deep cortex (layer VI) (5100 x 925 μm^2^), in areas remote from the area of densest amyloid burden.

### Inter-rater reliability of Aβ plaque subtype quantitation

The number and subtype of Aβ plaques and non-plaque associated regions of astrogliosis were quantitated in the area of densest burden in a subset of 10 randomly selected cases by two independent observers (RM and JES), and the extent of agreement assessed by calculating Gwet’s AC2 coefficients [45]. The coefficient calculations were performed using Agreestat 2011.2 programme (Advanced Analytics, Gaithersburg, MD, USA), and the extent of agreement was assessed using the benchmark proposed by Landis and Koch [46], a coefficient >0.6 indicating substantial agreement and a value >0.8 near-perfect agreement.

There was near-perfect agreement in the scoring of diffuse plaques with no associated astrocytes (AC2 = 0.86, 95%CI(AC2) 0.62; 1.00), diffuse plaques with focal astrogliosis (AC2 = 0.88, 95%CI(AC2) 0.75; 1.00), compact plaques with no associated astrocytes (AC2 = 0.83, 95%CI(AC2) 0.61; 1.00), compact plaques with circumferential astrogliosis (AC2 = 0.86, 95%CI(AC2) 0.65; 1.00) and large areas of non-plaque associated astrogliosis (AC2 = 0.93, 95%CI(AC2) 0.76; 1.00). There was a substantial agreement in the scoring of diffuse plaques with circumferential astrogliosis (AC2 = 0.76, 95%CI(AC2) 0.36; 1.00), compact plaques with focal astrocytes (AC2 = 0.79, 95%CI(AC2) 0.42; 1.00) as well as small areas of non-plaque associated astrogliosis (AC2 = 0.75, 95%CI(AC2) 0.29; 1.00), confirming the reliability of the scoring method. Subsequent analyses of the number and subtype of Aβ plaques and non-plaque associated regions of astrogliosis in the cohort were performed on scores by RM.

### Statistical Analysis

Statistical analyses were performed and graphs obtained using IBM SPSS Statistics 21 (Armonk, NY) and Stata Statistical Software 12 (College Station, TX). The association between Braak and Braak neurofibrillary tangle stage and the frequency of either diffuse or compact Aβ plaques was assessed using Kendall’s tau correlation coefficient. The relationships between plaque subtype with Braak and Braak stage and MMSE score were tested via multiple linear regression analysis where MMSE score and Braak and Braak stage were the dependent variables, whereas the relationships between plaque subtype with dementia status and *ApoE* genotype were verified using logistic regression, where dementia status and E4 ApoE genotype were the dependent variables. All the regression analyses were controlled by sex and age at death.

## Results

### Aβ plaque subtypes in the ageing brain

Diffuse and compact Aβ plaques were diverse in size, number and distribution throughout the cohort. The frequency of each grade (none, mild, moderate or severe) of diffuse and compact Aβ amyloid plaque in the cingulate gyrus is shown in Table 2. Both diffuse (τ = 0.333, p<0.001) and compact (τ = 0.259, p = 0.001) Aβ plaques associated with Braak and Braak neurofibrillary tangle stage.

**Table 2:** Frequency of diffuse and compact Aβ plaques in the cingulate gyrus, based on the CERAD protocol.

	Grade	Frequency (no of cases)	Percentage of cases
Diffuse Aβ plaques	None	13	11.9
	Mild	26	23.9
	Moderate	29	26.6
	Severe	41	37.6
Compact Aβ plaques	None	29	26.6
	Mild	43	39.4
	Moderate	37	33.9

**Fig 1:**
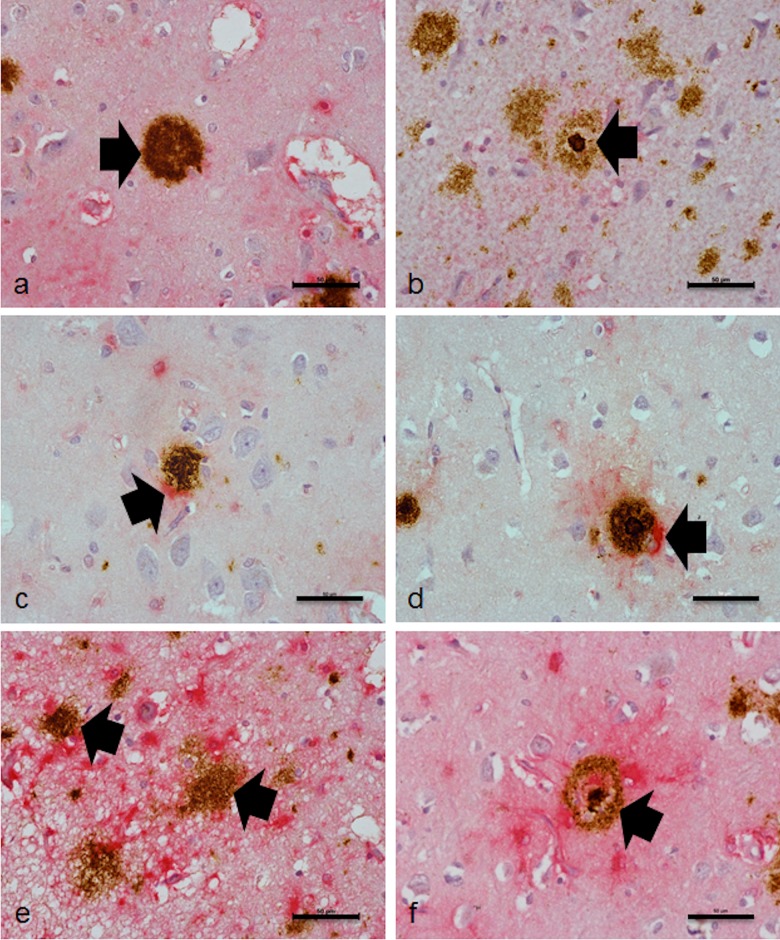
Aβ plaque subtypes in the ageing brain. Six distinct Aβ plaque subtypes were identified in the ageing cohort: (a) diffuse plaques with no associated astrocytes; (b) compact plaques (also known as classical or core plaques) with no associated astrocytes; (c) diffuse plaques with focal astrogliosis; (d) compact plaques with focal astrogliosis; (e) diffuse plaques with circumferential astrogliosis; (f) compact plaques with circumferential astrogliosis, as indicated by the arrow. Scale bar represents 50μm

**Fig 2:**
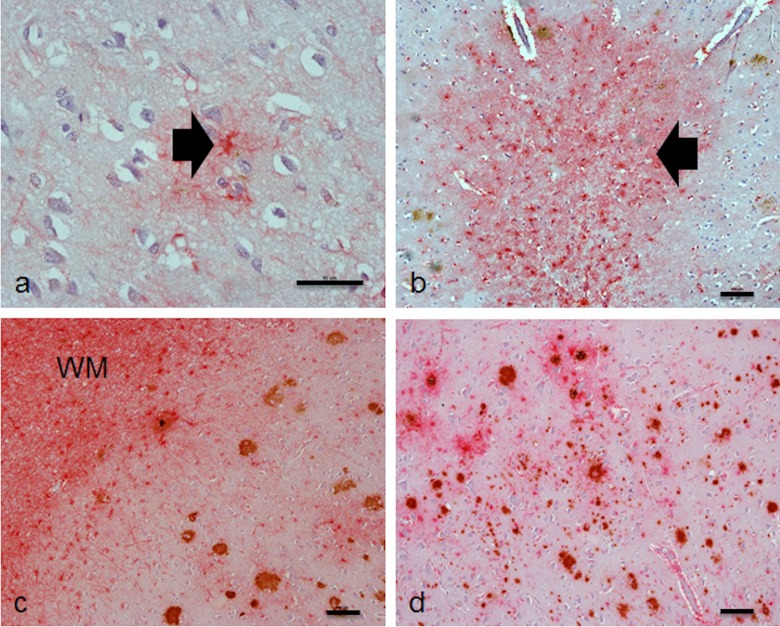
Astrogliosis remote from Aβ plaques, Aβ plaques in layer VI and regions of densest plaque burden. Both (a) small and (b) large areas of astrogliosis were detected in regions remote from Aβ plaques, as indicated by the arrow. (c) Clusters of plaques with associated reactive astrocytes were frequently observed in the deep cortex (layer VI) near the white matter border (WM), distinct from (d) the areas of densest amyloid burden. Scale bar represents 50μm (a) and 100μm (b-d).

Six distinct Aβ plaque subtypes were identified in the ageing cohort: (1) diffuse plaques with no associated astrocytes (Fig. 1A); (2) compact plaques with no associated astrocytes (Fig. 1B); (3) diffuse plaques with focal astrogliosis (Fig. 1C); (4) compact plaques with focal astrogliosis (Fig. 1D); (5) diffuse plaques with circumferential astrogliosis (Fig. 1E); (6) compact plaques with circumferential astrogliosis (Fig. 1F).

Both small (Fig. 2A) and large areas of astrogliosis (Fig. 2B) were also detected in regions remote from plaques. Initial investigation of the cohort noted that clusters of plaques with associated reactive astrocytes were frequently observed in the deep cortex near the white matter border (layer VI) (Fig. 2C), distinct from the areas of densest amyloid burden in layers I-V (Fig. 2D), therefore Aβ plaque subtype was assessed in both regions.

### Association of Aβ plaque subtype with Braak stage

In the area of densest burden diffuse plaques with no astrogliosis (β = 0.01, p<0.001) or with focal astrogliosis (β = 0.07, p<0.001) significantly associated with increasing Braak and Braak neurofibrillary tangle stage (Table 3). In layer VI, diffuse plaques with no (β = 0.04, p<0.001), focal (β = 0.08, p<0.001) or circumferential astrogliosis (β = 0.2, p = 0.003), as well as compact plaques with no (β = 0.21, p = 0.001), focal (β = 0.38, p<0.001) or circumferential astrogliosis (β = 0.45, p = 0.001) significantly associated with Braak stage (Table 4). Large areas (β = -0.32, p = 0.043), but not small areas (β = 0.02, p = 0.413), of non-plaque associated astrogliosis significantly associated with Braak stage.

**Table 3:** Association of Aβ plaque subtype in the area of densest Aβ burden with Braak stage, general cognition (MMSE), dementia status and possession of ApoEε4 allele.

	Braak^a^ (n = 101)			MMSE^a^ (n = 107)			Dementia^b^ (n = 107)			ApoEε4^b^ (n = 83)		
Aβ plaque subtype	β	95%CI(β)	p	β	95%CI(β)	p	OR	95%CI(OR)	p	OR	95%CI(OR)	p
*Diffuse plaque*												
No astrogliosis	0.04	(0.02; 0.06)	<0.001	-0.05	(-0.08; -0.02)	0.001	1.01	(1.00; 1.01)	0.187	1.02	(1.01; 1.03)	0.001
Focal astrogliosis	0.08	(0.03; 0.12)	<0.001	-0.27	(-0.47; -0.07)	0.009	1.06	(0.99; 1.14)	0.081	1.10	(1.02; 1.19)	0.016
Circumferential astrogliosis	0.20	(0.07; 0.32)	0.003	-0.36	(-1.95; 1.24)	0.659	1.13	(0.74; 1.74)	0.567	1.33	(0.87; 2.03)	0.187
*Compact plaque*												
No astrogliosis	0.21	(0.09; 0.33)	0.001	-0.73	(-1.59; 0.14)	0.100	1.09	(0.88; 1.35)	0.447	1.07	(0.85; 1.36)	0.559
Focal astrogliosis	0.38	(0.21; 0.55)	<0.001	-0.38	(-2.11; 1.34)	0.659	1.63	(0.96; 2.76)	0.071	1.26	(0.81; 1.94)	0.302
Circumferential astrogliosis	0.45	(0.18; 0.72)	0.001	-1.46	(-4.04; 1.12)	0.265	1.21	(0.53; 2.77)	0.648	1.11	(0.60; 2.04)	0.744

^a^multiple linear regression analyses used with MMSE score and Braak and Braak stage as dependent variables (test statistic: regression coefficient: β, beta).

^b^logistic regression analyses used dementia status and ApoE genotype as dependent variables (test statistic: odds ratio (OR) CI: confidence interval.

**Table 4:** Association of Aβ plaque subtype in the deep cortex (layer VI) with Braak stage, general cognition (MMSE), dementia status and possession of ApoEε4 allele.

	Braak^a^ (n = 101)			MMSE^a^ (n = 107)			Dementia^b^ (n = 107)			ApoEε4^b^ (n = 83)		
Aβ plaque subtype	β	95%CI(β)	p	β	95%CI(β)	p	OR	95%CI(OR)	p	OR	95%CI(OR)	p
*Diffuse plaque*												
No astrogliosis	0.01	(0.01; 0.02)	<0.001	-0.15	(-0.28; -0.03)	0.017	1.05	(1.01; 1.10)	0.025	1.04	(1.00; 1.07)	0.064
Focal astrogliosis	0.07	(0.04; 0.10)	<0.001	-0.15	(-0.43; 0.13)	0.288	1.09	(1.00; 1.19)	0.061	1.08	(1.00; 1.17)	0.054
Circumferential astrogliosis	0.21	(-0.04; 0.45)	0.093	0.04	(-0.82; 0.89)	0.934	1.06	(0.84; 1.33)	0.624	1.17	(0.95; 1.45)	0.141
*Compact plaque*												
No astrogliosis	0.12	(-0.01; 0.25)	0.064	-0.81	(-1.54; -0.08)	0.030	1.21	(0.93; 1.58)	0.154	1.77	(1.21; 2.59)	0.003
Focal astrogliosis	0.14	(-0.14; 0.41)	0.326	-0.64	(-1.76; 0.48)	0.261	1.29	(0.93; 1.79)	0.120	1.50	(1.09; 2.07)	0.014
Circumferential astrogliosis	0.03	(-0.37; 0.42)	0.892	-1.24	(-3.07; 0.58)	0.181	1.16	(0.71; 1.89)	0.552	1.13	(0.70; 1.82)	0.619

^a^multiple linear regression analyses used with MMSE score and Braak and Braak stage as dependent variables (test statistic: regression coefficient: β, beta).

^b^logistic regression analyses used dementia status and ApoE genotype as dependent variables (test statistic: odds ratio (OR) CI: confidence interval.

### Aβ plaques with little or no associated astrogliosis correlate with cognitive impairment

In the area of densest burden, diffuse Aβ plaques with no astrogliosis (β = -0.05, p = 0.001) or with focal astrogliosis (β = -0.27, p = 0.009) significantly associated with lower MMSE scores (Table 3). In layer VI, both diffuse plaques and compact plaques without astrogliosis significantly associated with lower MMSE scores (β = -0.15, p = 0.017 and β = -0.81, p = 0.03, respectively) (Table 4). Only diffuse plaques with no astrogliosis in the deep cortex significantly related to dementia status (p = 0.025) (Table 3). Neither small nor large areas of non-plaque associated astrogliosis associated with either MMSE scores (β = -0.24, p = 0.114 and β = -0.25, p = 0.791, respectively) or dementia status (p = 0.248 and p = 0.558).

### Association of Aβ plaque subtype with ApoE genotype

Possession of at least one *ApoE*ε4 allele was significantly associated with a greater number of diffuse plaques with no (OR = 1.02, p = 0.001) or focal astrogliosis (OR = 1.10, p = 0.016) in the region of densest burden (Table 3), and with compact plaques with no (OR = 1.77, p = 0.003) or focal astrogliosis (OR = 1.50, p = 0.014) in layer VI (Table 4). Neither small (OR = 0.92, p = 0.313) nor large (OR = 0.89, p = 0.693) areas of non-plaque associated astrogliosis associated with ApoE genotype. A summary of the major Aβ plaque subtype associations with Braak stage, general cognition (MMSE) and possession of the ApoEε4 allele is shown in Table 5.

**Table 5:** Summary of the major Aβ plaque subtype associations with Braak stage, general cognition (MMSE) and possession of the ApoEε4 allele.

Diffuse Aβ plaque				
Associated astrocytes	none		focal	
	densest burden	Layer VI	densest burden	Layer VI
increasing Braak stage	**<0.001**	**<0.001**	**<0.001**	**<0.001**
decreasing MMSE	**0.001**	**0.017**	**0.009**	0.288
possession of ApoEε4	**0.001**	0.064	**0.016**	0.054
**Compact Aβ plaque**				
Associated astrocytes	none		focal	
	densest burden	Layer VI	densest burden	Layer VI
increasing Braak stage	**0.001**	0.064	**<0.001**	0.326
decreasing MMSE	0.100	**0.030**	0.659	0.261
possession of ApoEε4	0.559	**0.003**	0.302	**0.014**

Diffuse Aβ plaques with no or focal astrogliosis in the area of densest burden associated with increasing Braak stage, decreasing MMSE and possession of at least one ApoEε4 allele. Diffuse Aβ plaques with no astrogliosis in layer VI associated with increasing Braak stage and decreasing MMSE score. Diffuse plaques with focal astrogliosis associated with increasing Braak stage. Compact Aβ plaques with no or focal astrogliosis in the area of densest burden associated with Braak stage. Compact Aβ plaques with no astrogliosis in layer VI associated with a decreasing MMSE score. Compact Aβ plaques with no or focal astrogliosis in layer VI associated with possession of at least one ApoEε4 allele.

## Discussion

Several studies have demonstrated an association between Aβ plaques and astrogliosis; however whether these reactive astrocytes are actively contributing to ongoing neurodegenerative processes or play a neuroprotective role is highly debated [11,13,14,47]. The results of the current study demonstrate that astrogliosis associated with both diffuse and compact plaques in the area of densest burden and in the deep cortex (layer VI) negatively relates to cognitive impairment, and that possession of at least one copy of the *ApoEε4* allele impacts the astroglial response to Aβ plaques.

The amyloid cascade hypothesis is currently the major theory of AD pathogenesis [48], however therapies based on removal of A have, to date, been disappointing [49]. Population-based studies have shown a weak association of amyloid pathologies with dementia [36,50], suggesting other factors contribute to cognitive impairment in the ageing brain. An additional possibility may be population variation in the response to Aβ deposits, with some individuals better able to prevent toxic effects on the surrounding neuropil. Inter-individual variation in the astrocyte response to Aβ deposition may be a part of this, and furthermore, ApoE genotype may be one regulator of the astrocyte response.

Amyloid plaque development starts in the superficial layers of the cortex and extends to the deep cortex as pathology progresses [51]. Increased levels of diffuse plaques without astrogliosis, but not plaque number, in both areas of densest burden and cortical layer VI demonstrated a significant association with lower MMSE scores, suggesting astrocytes play a neuroprotective role when associated with amyloid deposits. Diffuse, but not compact, plaques with no astrogliosis in layer VI strongly associated with dementia status.

In contrast to compact plaques which contain the fibrillar form of Aβ, diffuse plaques contain pre-fibrillary Aβ and may represent a precursor in plaque development [52]. The different composition of plaques may result in differences in toxicity, as intermediate forms of amyloid are considered as one of the most neurotoxic species of Aβ [1]. Intraneuronal accumulation of Aβ, rather than extracellular Aβ deposition may contribute to neuronal dysfunction and drive AD pathology [53–55]. Future studies aimed at specifically assessing the association between astrogliosis and intraneuronal Aβ are required. We cannot demonstrate how reactive astrocytes might promote neuronal survival but they have been shown to protect neurones by regulating extracellular ion concentrations and neurotransmitter recycling [56], secreting neurotrophic factors [57], and to modulate Aβ-mediated neurotoxicity *in vitro*, safeguarding against neuronal dystrophy and synaptic loss [58]. Furthermore, astrocytes can degrade, internalise and clear Aβ [13,28,59], and have been shown to regulate microglial phagocytosis of compact plaque cores [20]. Our data suggests that astrocytes may form a protective barrier around amyloid plaques, demarcating the area for Aβ degradation, phagocytosis and a local inflammatory reaction. This would predict that plaques that are not insulated by astrocytes have a greater toxic effect on surrounding brain tissue. However, further investigations are required to confirm and characterise the neuroprotective role of astrocytes in response to Aβ plaque formation.

In the area of densest amyloid burden diffuse plaques with no, or focally, associated reactive astrocytes, but not with circumferential astrogliosis, demonstrated a significant association with Braak stage while all compact plaque subtypes showed a significant association with Braak stage, confirming the increasing burden of amyloid plaques in the cingulate gyrus mirrors the progression of tau pathology in the ageing brain [60]. The lack of association between diffuse plaques with circumferential astrogliosis and Braak stage suggests that the astrocyte reaction to plaque formation occurs at the earliest stages of tangle pathology, and does not directly parallel amyloid deposition [11]. Large regions of non-plaque associated astrogliosis showed no relation to dementia status or cognitive impairment, but did significantly associate with increasing Braak stage, supporting studies which suggest these astrogliotic lesions parallel neurofibrillary tangle progression and react to the burden of neurofibrillary tangles in the ageing brain [11,61]. AD is characterised by early damage to synapses [62,63] and dendritic atrophy [64]. Further work is required to investigate if the regions of non-plaque associated astrogliosis detected in this study reflect astrocyte reaction to dendritic degeneration and synaptic loss [60,65].

Possession of a single copy of the *ApoEε4* allele is associated with a significant increased risk of developing AD [21,26], increased numbers of Aβ plaques [24], increased accumulation of intraneuronal Aβ [66] and elevated levels of astrogliosis [67]. In contrast to previous reports that ApoEε4 genotype does not impact glial responses to plaques in AD studies [47], the current findings demonstrate that in the area of densest Aβ burden diffuse amyloid plaques with little or no associated astrogliosis are significantly higher in ApoEε4 carriers in the ageing population. Although studies have demonstrated a significant correlation between compact plaques and ApoE genotype in AD [68,69], only compact plaques with no or focal astrogliosis in layer VI correlated with ApoE genotype. We have previously shown that astrocyte dysfunction in association with the progression of Alzheimer-type pathology is an early event for ApoEε4 carriers in this ageing cohort [25], and propose that this ApoEε4-associated astrocyte dysfunction may explain the lack of association with plaques with circumferential astrogliosis, and the significant association between increased levels of plaques with no or little astrogliosis and ApoE genotype.

In addition to an astrocyte response to Aβ plaques, microglial activation is also a prominent feature of AD pathology [70,71], and is associated with the degradation and clearance of Aβ [72,73]. While the activation and recruitment of microglia may occur in tandem with astrogliosis, studies have shown that reactive astrocytes and activated microglia respond differently to Aβ plaque formation and development [47], with activated microglia associated with proliferation and the secretion of pro-inflammatory cytokines [74]. Furthermore, CNS injury is associated with crosstalk between astrocytes and microglia involving a cytokine network, which regulates glial activation and impacts neuronal survival [75]. Future studies assessing microglial activation in addition to astrogliosis will enable a detailed characterisation of the glial response to Aβ plaque formation in the ageing brain.

The current study examined the astrocyte response to amyloid plaques solely in the cingulate gyrus, a region associated with metabolic and vascular changes in the very early stages of AD [33,34]. Expanding the investigation to include additional brain regions is essential to provide further validation to the findings reported here. In this study, the detection of plaques without associated astrocytes in a single section may have failed to detect a focal astrocyte response associated with larger Aβ plaques which span several sections. Quantitation of the number and subtype of plaques in serial sections, as opposed to a single field, would enable a three-dimensional astrocyte response to Aβ plaques to be determined. Further investigation into areas of reactive astrocytes remote from Aβ plaques should be performed to enable clearer definition of these lesions with respect to other local pathological features including synaptic loss, dendritic atrophy, tau pathology and microglial activation, as discussed above.

The current population-based study of the astrocyte response to amyloid plaques demonstrates clear relationships between Aβ plaque subtypes and cognitive impairment. Our findings may indicate a neuroprotective role of plaque-associated astrocytes, and suggest that astrogliosis may attenuate the neurotoxic effects of Aβ in the ageing brain. These findings encourage future studies to confirm the neuroprotective role of plaque-associated astrocytes and elucidate the precise mechanism(s) which may aid in the development of novel therapeutic strategies.

## Conclusions

Reactive astrocytes in close proximity to either diffuse or compact plaques may have a neuroprotective role in the ageing brain, and possession of at least one copy of the *ApoEε4* allele impacts the astroglial response to Aβ plaques.
